# Dysregulation of SIRT-1 in aging mice increases skeletal muscle fatigue by a PARP-1-dependent mechanism

**DOI:** 10.18632/aging.100696

**Published:** 2014-10-28

**Authors:** Junaith S. Mohamed, Joseph C. Wilson, Matthew J. Myers, Kayla J. Sisson, Stephen E. Alway

**Affiliations:** ^1^ Laboratory of Muscle Biology and Sarcopenia, Division of Exercise Physiology, West Virginia University School of Medicine, Morgantown, West Virginia 26506-9227, USA; ^2^ Center for Cardiovascular and Respiratory Sciences, West Virginia University School of Medicine, Morgantown, West Virginia 26506-9227, USA; ^3^ West Virginia Clinical and Translational Science Institute, West Virginia University School of Medicine, Morgantown, West Virginia 26506-9227 USA

**Keywords:** Aging, oxidative stress, PARP-1, sarcopenia, SIRT-1, skeletal muscle

## Abstract

Accumulation of reactive oxygen species (ROS) in skeletal muscles and the resulting decline in muscle performance are hallmarks of sarcopenia. However, the precise mechanism by which ROS results in a decline in muscle performance is unclear. We demonstrate that isometric-exercise concomitantly increases the activities of Silent information regulator 1 (SIRT-1) and Poly [ADP-ribose] polymerase (PARP-1), and that activated SIRT-1 physically binds with and inhibits PARP-1 activity by a deacetylation dependent mechanism in skeletal muscle from young mice. In contrast, skeletal muscle from aged mice displays higher PARP-1 activity and lower SIRT-1 activity due to decreased intracellular NAD^+^ content, and as a result reduced muscle performance in response to exercise. Interestingly, injection of PJ34, a PARP-1 inhibitor, in aged mice increased SIRT-1 activity by preserving intracellular NAD^+^ content, which resulted in higher skeletal muscle mitochondrial biogenesis and performance. We found that the higher activity of PARP-1 in H_2_O_2_-treated myotubes or in exercised-skeletal muscles from aged mice is due to an elevated level of PARP-1 acetylation by the histone acetyltransferase General control of amino acid synthesis protein 5-like 2 (GCN-5). These results suggest that activation of SIRT-1 and/or inhibition of PARP-1 may ameliorate skeletal muscle performance in pathophysiological conditions such as sarcopenia and disuse-induced atrophy in aging.

## INTRODUCTION

Sarcopenia is an advanced age-related loss of skeletal muscle and function, which reduces the amount of metabolically active tissue, thus, increasing the risk for metabolic diseases [1, 2], and negatively impacts mobility, limiting the independence living and quality of life of elderly individuals [3, 4]. The course of sarcopenia and age-related diseases that are associated with sarcopenia [1, 5] involve complex processes that are controlled by both extrinsic and intrinsic factors, many of which converge on a decline in the ability of muscle stem cells (satellite cells) to replace and repair damaged muscle fibers in old hosts [6-8].

Although the mechanisms that initiate of sarcopenia are largely unknown, an increased production and accumulation of reactive oxygen species (ROS) has been proposed to underlie the pathogenicity of sarcopenia [9, 10]. Oxidative stress occurs when the production of oxidants exceeds the capacity of the cells to eliminate or buffer oxidizing reactions to proteins, DNA and lipids. The elevated levels of oxidized molecules may contribute to the progression of sarcopenia by controlling many cellular pathways including redox-sensitive signaling pathways [11]. The mitochondria are both ROS producers and are adversely affected by excessive ROS levels. For example, a comparative electron-microscopic study of the ultrastructure of mitochondria in skeletal muscles of young (low basal ROS levels) and old rats (high basal levels of ROS) revealed age-dependent changes in both the general organization of the mitochondrial reticulum and the ultrastructure of mitochondria [12], which presumably contributes to the age-associated dysfunction of this organelle. Furthermore, early treatment of aged mice with the mitochondrial antioxidant SkQ1 was shown to prevent the development of age-dependent destructive pathological changes in mitochondria [12].

One group of factors sensitive to changes in the cellular redox pathways is the poly(ADP-ribose) polymerases (PARPs). ROS have a robust ability to induce the poly (ADP-ribosyl)ation (PARylation) of many proteins, which regulate the cell cycle, growth, and survival, thereby positioning PARylation as an important biochemical marker of oxidative stress [13].

PARylation is one of the post-translational modifications of proteins that are regulated by the PARP family of enzymes in most eukaryotic organisms [14] PARPs catalyze the covalent transfer of mono or poly(ADP) units from nicotinamide adenine dinucleotide (NAD^+^) to glutamate or aspartate residues within target proteins, resulting in the synthesis of a large chain of branched ADP-ribose polymers [15]. This post-translational modification either alters the functional properties of PAR-binding proteins or allows the proteins to be degraded by poly (ADP-ribose) glycohydrolase [16]. Surprisingly, many of the putative PAR-binding proteins regulate a wide range of cellular functions including cell survival [13]. PARP-1 is the most extensively studied PARP family protein that requires a source of nuclear NAD^+^ for its function [17, 18]. Although basal activation of PARP-1 is necessary to maintain normal cell homeostasis, over activation of PARP-1 by ROS species such as superoxide (O_2_^−^) and hydrogen peroxide (H_2_O_2_) increases protein PARylation and depletes intracellular NAD^+^ levels leading to cell death [19]. This effect suggests that tight regulation of PARP-1 activity is important for cell survival.

Many lines of evidence have shown that caloric restriction is an effective intervention to slow the aging process in most organisms, and thereby delaying the onset of age-related disease and functional decline [20, 21] Although a wide range of signaling pathways regulates the effects of caloric restriction on aging, Silent information regulator 1 (SIRT-1) has emerged as a promising target from these pathways [22-26]. SIRT-1 has been shown to inhibit the differentiation of mouse C2C12 myoblasts and reduce the expression of myogenin, which is an important regulator for the myogenic specification and differentiation of activated satellite cells [27, 28]. Furthermore, SIRT-1 has been shown to directly induce proliferation of satellite cells [29]. These findings suggest that SIRT-1 may have an important role in prolonging or enhancing proliferation of satellite cells. However, while satellite cell function is reduced with aging, and thus, lower SIRT-1 protein levels might be expected in muscles of old animals, it is interesting to note that increased levels of SIRT-1 have been reported in satellite cells isolated from old rats [30], although the significance of this is not clear. One possibility to explain this complex role of SIRT-1 in skeletal muscle is that SIRT-1 activity and not the abundance of this protein may be more important for determining the downstream function in sarcopenic muscle as it is in muscles of young animals [31]. Thus, understanding the pathways that regulate the activity of SIRT-1 is critical, especially in aging muscles.

SIRT-1 is an NAD^+^-dependent protein deacetylase, modulation of nuclear NAD^+^ levels can alter the activity of SIRT-1 in skeletal muscle [32, 33], which is presumably independent of any changes in protein levels occurring with aging. However, high cellular NAM and/or NADH levels inhibit SIRT-1 activity [34], suggesting that increasing cellular NAD^+^ levels would be an effective way to activate the SIRT-1 pathway in aging muscle. SIRT-1 and PARP-1 regulate many common pathways, including oxidative stress responses and cell survival. Furthermore, SIRT-1 and PARP-1 compete for the same NAD^+^ pool. As a result, if PARP-1 utilizes NAD^+^ at a high rate to increase its activity, we would anticipate that SIRT-1 will have less NAD^+^ available to it, which in turn should suppress the activity of SIRT-1. [32, 35]. This idea is consistent with observations showing that activation of PARP-1 upon cellular stress depletes intracellular NAD^+^ stores and subsequently releases high levels of NAM, which in turn, significantly inhibits SIRT-1 activity [32, 36, 37]. These findings suggest that these two proteins might be able to counterbalance each other's activity to regulate cell survival. However, the mechanism by which SIRT-1 inhibits the activity of PARP-1 and protects skeletal muscles from ROS-induced decline in muscle performance is unknown. We show here that exercise increases the activity of SIRT-1, which physically binds to and inhibits PARP-1 activity via a deacetylation-dependent mechanism in skeletal muscles from young mice. This protective role of SIRT-1 dramatically declined in skeletal muscles from aged mice as a result of PARP-1 over-activation. Pharmacological inhibition of PARP-1 restored the effect of SIRT-1, and as a result increased skeletal muscle performance in aged mice. Moreover, the enhanced-activity of PARP-1 in skeletal muscles from aged mice is due to an elevated level of acetylation of PARP-1 by General control of amino acid synthesis protein 5-like 2 (GCN5). These results suggest that activation of SIRT-1 and/or inhibition of PARP-1 could be a therapeutic strategy for the treatment of the age-associated decline in muscle performance and potentially counterbalance the functional decay in sarcopenia.

## RESULTS

### SIRT-1 protects skeletal muscle in young mice from PARP-1 by deacetylation-dependent mechanism

**Figure 1:**
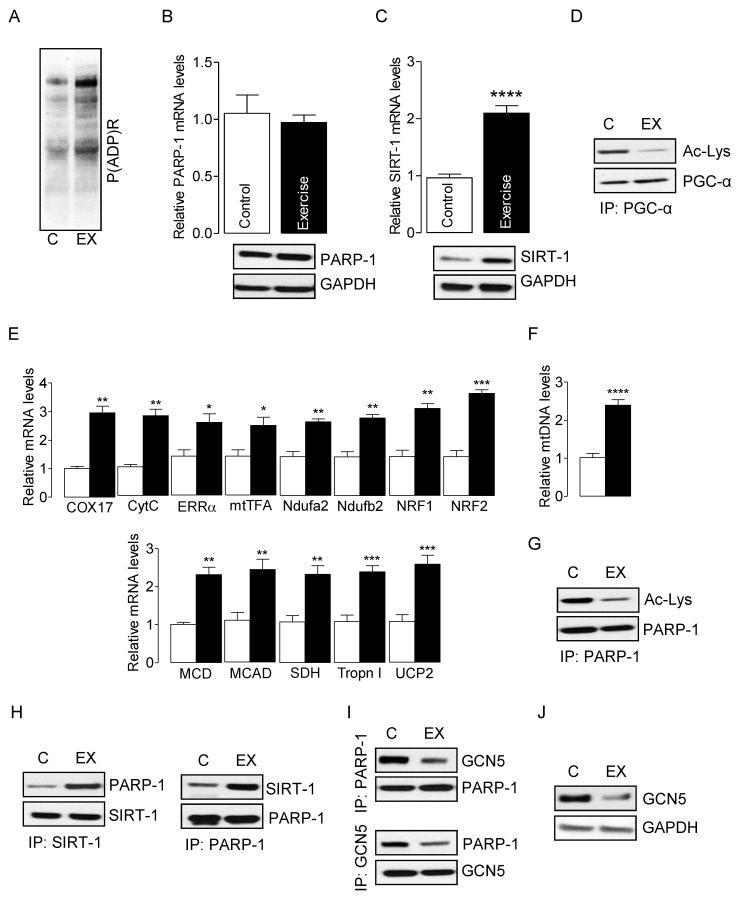
SIRT-1 deactivates exercise-induced PARP-1 in skeletal muscle from young mice Total RNA and cell lysates were isolated from the control or exercised gastrocnemius muscle. (**A**) Global cellular protein PARylation was determined in total cell lysates by immunoblots. PARP-1 (**B**) and SIRT-1 (**C**) mRNA (top) and protein (bottom) levels were determined in total muscle mRNA and cell lysate, respectively. GAPDH was used as a loading control. (**D**) PGC-1α acetylation levels were estimated by immunoblotting after IP. (**E**) mRNA expression of the indicated genes in the total RNA was examined by qPCR. (**F**) mtDNA was evaluated in total muscle genomic DNA by qPCR. (**G**) PARP-1 acetylation levels were estimated by immunoblotting after IP. (**H**) SIRT-1 and PARP-1 or (I) GCN5 and PARP-1 binding assays were estimated by immunoblotting after IP. (**J**) GCN5 protein levels were determined in total cell lysates by immunoblots. The blots are representative of three independent experiments. The data are presented as mean ± SEM (n = 3). White and black bars indicate non-exercised and exercised gastrocnemius muscle, respectively. COX17, cyclooxygenase 17; CytC, Cytochrome C; ERR-α, estrogen-related receptor α; mtTFA, mitochondrial transcription factor A; Ndufa2, NADH dehydrogenase [ubiquinone] iron-sulfur protein a 2; NRF1,nuclear respiratory factor 1; MCD, medium-chain acyl-CoA dehydrogenase; MCAD, medium-chain acyl-CoA dehydrogenase; SDH, succinate dehydrogenase; Tropn I, troponin I; UCP2, uncoupling protein 2; immunoprecipitation (IP).

To study the interplay between SIRT-1 and PARP-1 *in vivo*, we induced oxidative stress in skeletal muscles of young mice using electrically evoked isometric-exercise, which has been shown to generate ROS in skeletal muscle [9, 38]. Electrically evoked exercise modestly increased PARP-1 activity in skeletal muscles, as evidenced by global protein PARylation (Fig. 1A) without altering the mRNA and protein levels of PARP-1 (Fig. 1B). In contrast, exercise significantly increased SIRT-1 mRNA and protein levels (Fig. 1C) as well as SIRT-1 activity, as evidenced by PGC-1α hypo-acetylation (Fig. 1D). The increased activation of PGC-1α resulted in up-regulation of the genes necessary for mitochondrial biogenesis and oxidative metabolism in exercised-muscle (Fig. 1E). The increased mtDNA content was confirmative of an elevation in mitochondrial biogenesis (Fig. 1F). PARP-1 activity can be modulated by its acetylation level [37, 39], and therefore, we explored the acetylation of PARP-1, as a potential mechanism that would explain the decline in PARP-1 activity in skeletal muscle from young mice. IP assays using cell lysates from exercised and non-exercised skeletal muscles demonstrated that exercise decreased the acetylation of PARP-1 (Fig. 1G), suggesting that the reduced activity of PARP-1 in exercised skeletal muscle might be due to hypo-acetylation of PARP-1. Because SIRT-1 can deacetylate non-histone proteins, and given that exercise increased the activity of SIRT-1 (Fig. 1C and D), we sought to determine if SIRT-1 could deacetylate PARP-1. Data from IP assays showed an increased association between SIRT-1 and PARP-1 proteins in exercised muscles as compared to intra-animal non-exercised muscles (Fig. 1H). Earlier studies have shown that PCAF and p300/CBP are the acetyltransferases of PARP-1 [37, 39]. Therefore, we sought to determine whether the decreased acetylation of PARP-1 was due to a reduced association between PARP-1 and PCAF or p300/CBP. Surprisingly, we could not detect any significant association between PARP-1 and p300/CBP or PCAF (data not shown). However, we did observe a substantial interaction between PARP-1 and GCN5 in which electrically-evoked exercise significantly decreased the association between GCN5 and PARP-1 proteins (Fig. 1I). Concomitant with the decreased association of GCN5 with PARP-1, the level of GCN5 protein was also significantly lower in exercised-skeletal muscle (Fig. 1J). These results indicate that the potential mechanism whereby SIRT-1 protects skeletal muscle in young mice from the elevated PARP-1 activity that is seen in old age, is via increased deacetylation-dependent inactivation and/or reduced GCN5 levels in response to exercise.

### Dysregulation of SIRT-1 increases PARP-1 activity and reduces skeletal muscle performance in aged mice

**Figure 2:**
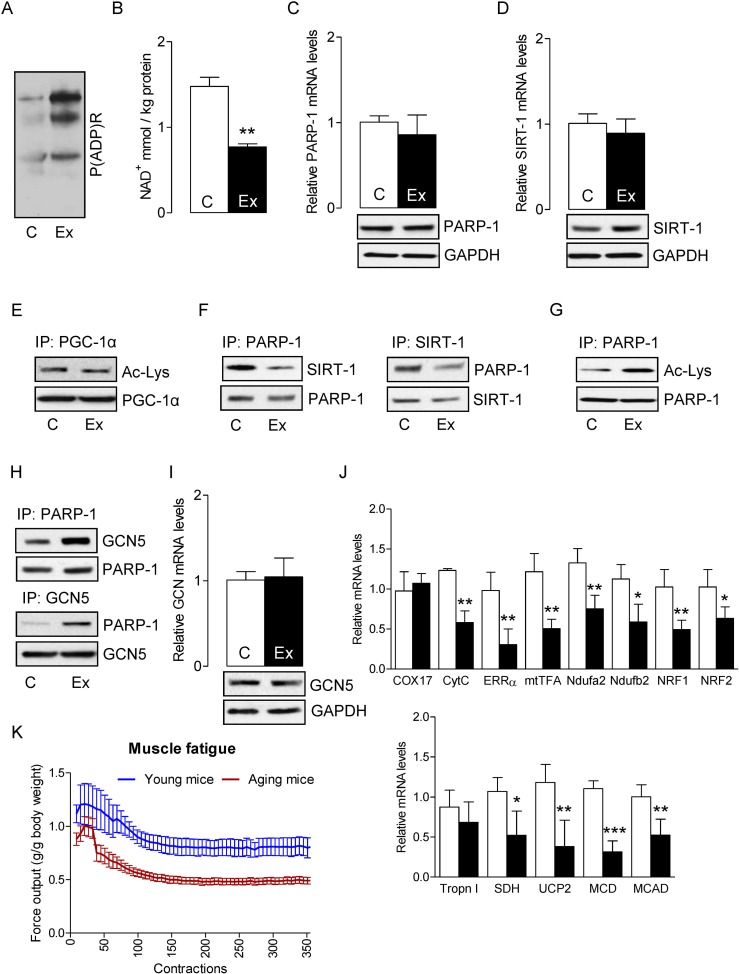
SIRT-1 dysregulation in aged mice increases skeletal muscle fatigue Total RNA and cell lysates were isolated from the control or exercised gastrocnemius muscles. (**A**) Global cellular protein PARylation was determined in total cell lysate by immunoblots. (**B**) NAD+ levels in skeletal muscle were determined in control and exercised muscles. PARP-1 (**C**) and SIRT-1 (**D**) mRNA (top) and protein (bottom) levels were determined in total muscle mRNA and cell lysates, respectively. GAPDH was used as a loading control. (**E**) PGC-1α acetylation levels were estimated by immunoblotting after IP. (**F**) SIRT-1 and PARP-1 binding assays, PARP-1 acetylation levels (**G**) or PARP-1 and GCN5 binding assays **(H**) were estimated by immunoblotting after IP. (**I**) GCN5 mRNA (top) and protein (bottom) levels were determined in total muscle mRNA and cell lysate, respectively. (**J**) mRNA expression of the indicated genes in the total RNA was examined by qPCR. (**K**) Maximal evoked isometric forces from 350 contractions are shown for the plantar flexor muscles in young and aged mice. All force measurements were normalized to body weight (g). The blots are representative of three independent experiments. The data are presented as mean ± SEM (n = 3). White and black bars indicate non-exercised and exercised gastrocnemius muscles, respectively. COX17, cyclooxygenase 17; CytC, Cytochrome C; ERR-α, estrogen-related receptor α; mtTFA, mitochondrial transcription factor A; Ndufa2, NADH dehydrogenase [ubiquinone] iron-sulfur protein a 2; NRF1,nuclear respiratory factor 1; MCD, medium-chain acyl-CoA dehydrogenase; MCAD, medium-chain acyl-CoA dehydrogenase; SDH, succinate dehydrogenase; Tropn I, troponin I; UCP2, uncoupling protein 2; immunoprecipitation (IP).

In skeletal muscles of aged mice, in which the basal oxidant level is already high, exercise has the ability to further increase oxidant production by as much as 80% [40]. As SIRT-1 inactivates PARP-1 and protects skeletal muscles in young mice, we sought to determine whether SIRT-1 could exert a similar effect in skeletal muscle from aged mice. Electrically evoked exercise robustly increased PARP-1 activity, as indicated by elevated global protein PARylation (Fig. 2A) and also reduced the intracellular NAD+ content (Fig. 2B) without changing PARP-1 mRNA and protein levels (Fig. 2C). Electrically-evoked exercise did not alter SIRT-1 mRNA and protein levels (Fig. 2D), or PGC-1α acetylation status, a result that was in contrast with the data from young mice (Fig. 2E). IP assays showed that the association between SIRT-1 and PARP-1 proteins in skeletal muscles from aged mice was greatly lower in response to exercise, which is in contrast to what we had observed for young mice (Fig. 2F). In agreement with these results, the acetylation level of PARP-1 was increased (Fig. 2G) due to an increased association of GCN5 with PARP-1 (Fig. 2H) in skeletal muscle from aged mice. To establish the role of PARP-1 acetylation by GCN5, we measured the mRNA and protein levels of GCN5. As illustrated in Fig. 2I, there were no changes in the levels of GCN5 mRNA and protein. As the acetylation status of PGC-1α was unchanged, it was not surprising to find that the genes that were necessary for mitochondrial biogenesis and oxidative metabolism in exercised-muscle were significantly lower in muscles from aged mice (Fig. 2J). To explore the dysregulation of SIRT-1 and over-activation of PARP-1 could change the muscle performance; we determined maximal isometric forces from the plantar flexor muscle of aged mice. As illustrated in Figure 2K, aged mice had an increased in vivo plantarflexor muscle fatigue as compared to young mice. This was shown by a shift to the right in the fatigue index after 30 contractions as compared to young mice. These results suggest that dysregulation of SIRT-1 in aged mice decreased mitochondrial biogenesis and oxide-tive metabolism, and increased PARP-1 activity, which in turn, increased muscle fatigue in response to exercise.

### Administration of the PARP-1 inhibitor PJ34 in aged mice increases SIRT-1- mediated mitochon-drial biogenesis and skeletal muscle performance

**Figure 3:**
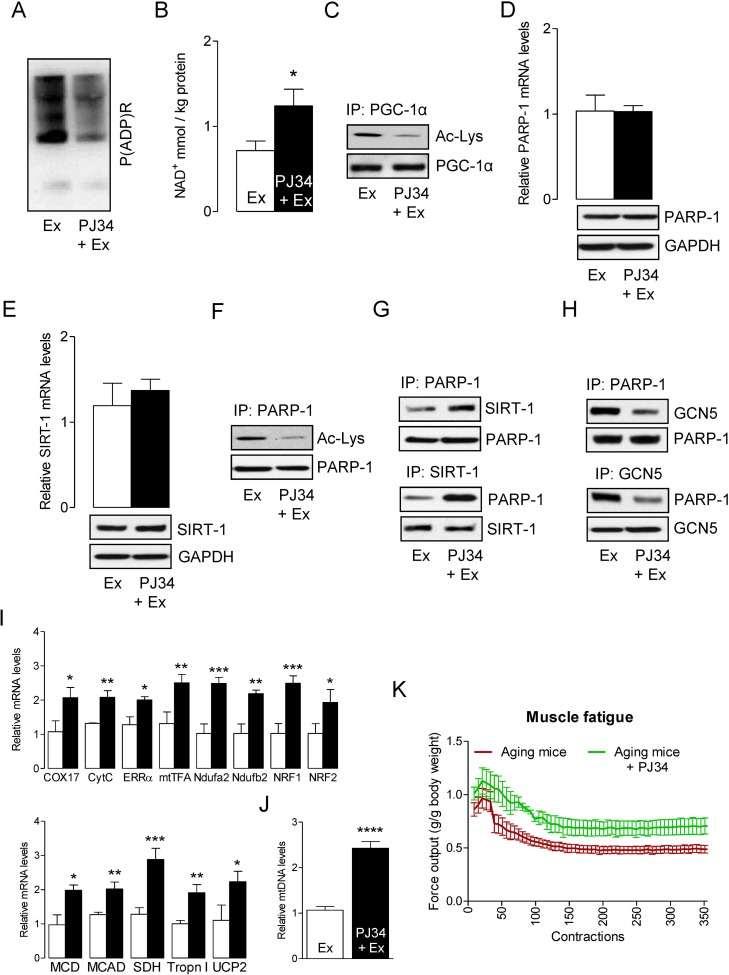
PARP-1 inhibition increases SIRT-1 activity and improves skeletal muscle fatigue Total RNA and cell lysates were isolated from the skeletal muscle of mice that were exercised and PBS injected (Ex) or exercised and PJ34 injected (Ex + PJ34). (**A**) Global cellular protein PARylation was determined in total cell lysates by immunoblots. (**B**) NAD^+^ levels were determined in in skeletal muscle of Ex or Ex + PJ34 mice. (C) PGC-1α acetylation levels were estimated by immunoblotting after IP. PARP-1 (**D**) and SIRT-1 (**E**) mRNA (top) and protein (bottom) levels were determined in total muscle mRNA and cell lysates, respectively. GAPDH was used as a loading control. PARP-1 acetylation levels (**F**), SIRT-1 and PARP-1 binding assays (**G**), or PARP-1 and GCN5 binding assays (**H**) were estimated by immunoblotting after IP. (I) mRNA expression of the indicated genes in the total RNA was examined by qPCR. (**J**) mtDNA was evaluated in total muscle genomic DNA by qPCR. (**K**) Maximal isometric forces from the plantar flexor muscles are presented for Ex or Ex + PJ34 mice. All force measurements were normalized to body weight (g). The blots are representative of three independent experiments. The data are presented as mean ± SEM (n = 3). White and black bars indicate Ex and Ex + PJ34 mice, respectively. COX17, cyclooxygenase 17; CytC, Cytochrome C; ERR-α, estrogen-related receptor α; mtTFA, mitochondrial transcription factor A; Ndufa2, NADH dehydrogenase [ubiquinone] iron-sulfur protein a 2; NRF1,nuclear respiratory factor 1; MCD, medium-chain acyl-CoA dehydrogenase; MCAD, medium-chain acyl-CoA dehydrogenase; SDH, succinate dehydrogenase; Tropn I, troponin I; UCP2, uncoupling protein 2. IP, immunoprecipitation.

We hypothesized that inhibition of PARP-1 by the PARP-1 inhibitor PJ34 would increase the activity of SIRT-1 by elevating intracellular NAD^+^. To test this hypothesis, we injected PJ34 intraperitoneally in aged mice (10 mg/kg bw) 24 h prior to exercise. In agreement with our hypothesis, inhibition of PARP-1 by PJ34 reduced the exercise-induced activation of PARP-1 (Fig. 3A) and this was followed by increasing the intracellular NAD^+^ content (Fig. 3B). Given the impact of PJ34 on the modulation of NAD^+^ levels, it was not surprising to observe that mice injected with the PARP-1 inhibitor displayed higher SIRT-1 activity, as demonstrated by reduced PGC-1α acetylation (Fig. 3C). However, inhibition of PARP-1 by PJ34 did not alter PARP-1 and SIRT-1 levels (Fig. 3D and E). The increased SIRT-1 activity coincided with a decreased acetylation of PARP-1 (Fig. 3F) that was due to a strong interaction between SIRT-1 and PARP-1, (Fig. 3G) and a weak interaction between GCN5 and PARP-1 (Fig. 3H). The increased activation of PGC-1α resulted in the up-regulation of genes that are necessary for mitochondrial biogenesis and oxidative metabolism in exercised-muscle (Fig. 3I). The increased content of mtDNA further confirmed the mitochondrial biogenesis (Fig. 3J). The initial rate of fatigue for the plantarflexors was reduced in PJ34-injected mice when compare to PBS injected mice (Fig. 3K). The initial force production in the PJ34 treated animals was ~ 11.3% greater than the PBS treated animals. Force typically increases for the first ~ 30 contractions in both animal groups, likely as a result of having enhanced calcium release, then force declined thereafter as fatigue occurred. However, the average percent of force decline (i.e. fatigue index= [contraction 1-contraction 50/contraction 1x100]) was greater in control (−15.4 ± 7.1%) than in PJ34 (−0.2 ± 8.4%) treated animals after the first 50 contractions, suggesting that PJ34 reduced the initial rate of fatigue thereby delaying the onset of fatigue. Similarly, the average fatigue index after 60 contractions was greater in PBS control as compared with PJ34 treated animals (−19.1 ±9.4% vs. −9.5 ± 6.9%). However, there were no differences between the rate of fatigue after 70 (−25.7 ±6.9% vs. −19.9 ± 7.6%), 80 (−18.8 ±6.7% vs. −14.4 ± 8.3%), 100, (−31.8 ±7.5% vs. −23.3 ± 7.1%), 125 (−20.4 ±4.1% vs. −27.5 ± 12.9%), or 150 (−39.2 ±8.1% vs. −29.8 ± 13.1%) contractions in control and PJ34 treated animals, respectively. Thus, while PJ34 appeared to delay fatigue by sustaining force production over the early series of contractions (<60 contractions), it did not improve the rate of fatigue per se after 60 contractions. Nevertheless, PJ34 did permit a greater total work (area under the force x time curve) to be performed over the course of the experiment such that PJ34 supplemented animals performed 34% more work at the beginning of the contractions, and this leveled off to ~20.4% more work by the 150^th^ contraction and stayed at this level throughout the remainder of the 360 contractions. Together these results suggest that the inhibition of PARP-1 may be an effective way to rescue skeletal muscles from ROS/PARP-1-induced decline in muscle performance (e.g., delay the onset of fatigue and increase the total sustained work) in sarcopenia.

### PARP-1 inhibition prevents ROS-induced myotube death via the SIRT-1 pathway

**Figure 4:**
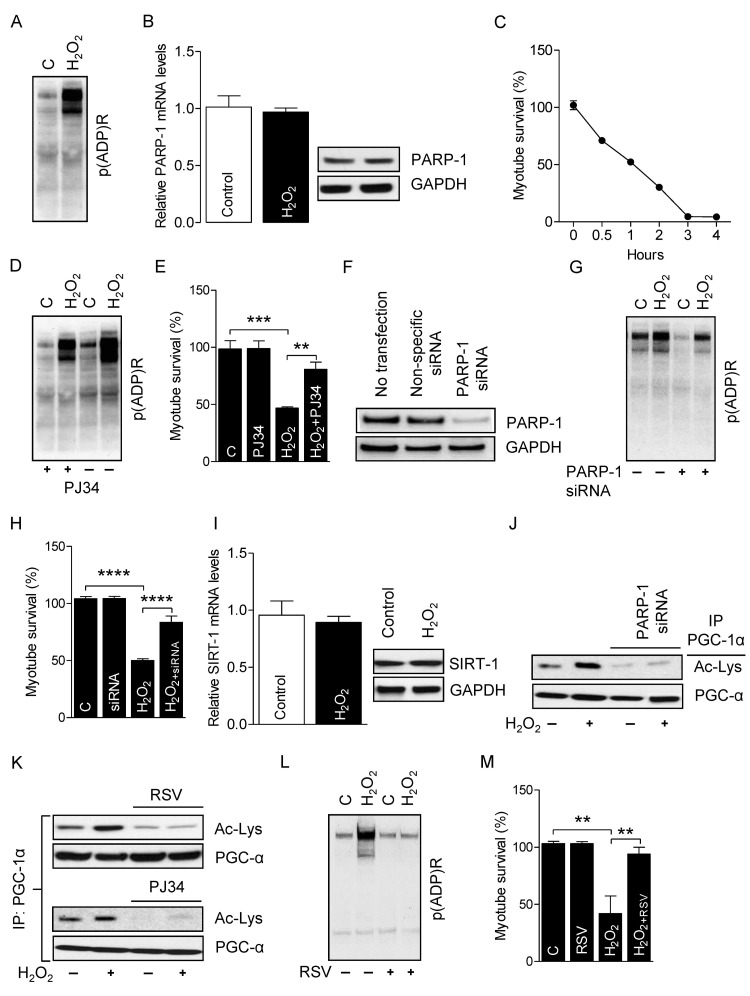
PARP-1 inhibition prevents the H2O2-induced myotube death Total RNA and cell lysates were isolated from myotubes either treated or not treated with H_2_O_2_. (**A**) Global cellular protein PARylation was determined in the total cell lysate by immunoblots. (**B**) PARP-1 mRNA and protein levels were determined in total mRNA and cell lysates, respectively. GAPDH was used as a loading control. (**C**) Myotube survival (%) in the presence or absence of H_2_O_2_ was determined by MTT assay at the indicated time-points. (**D**) Global cellular protein PARylation was determined in total cell lysates from either H_2_O_2_-treated or non-treated myotubes either in the presence or absence of PJ34 by immunoblots. (**E**) Myotube survival (%) was determined by the MTT assay in myotubes treated with the conditions similar to ‘D’. (**F**) Myotubes were transfected with non-specific or PARP-1-targeting siRNAs. PARP-1 protein abundance were analyzed 48 h after transfection by immunoblots. (**G**) Global cellular protein PARylation was determined in total cell lysates from either H_2_O_2_-treated or non-treated myotubes either in the presence or absence of PARP-1 by immunoblots. (**H**) Myotube survival (%) was determined by a MTT assay in myotubes treated with the conditions similar to ‘G’. (**I**) SIRT-1 mRNA (left side) and protein (right side) levels were determined in total mRNA and cell lysates, respectively. (J) PARP-1 acetylation levels were estimated from immunoblots after IP with the conditions similar to ‘G’. (**K**) PGC-1α acetylation levels were determined in total cell lysates from either myotubes treated with H_2_O_2_ or non-treated myotubes with or without PJ34 or RSV by immunoblotting after IP. Global cellular protein PARylation (**L**) or myotube survival (%) was determined in total cell lysates from either myotubes treated with H_2_O_2_ or non-treated myotubes with or without resveratrol. IP, immunoprecipitation; RSV, resveratrol.

Excessive ROS induces necrotic- and apoptotic-mediated cell death [41]. To determine the role of PARP-1 on the survival of myotubes, we incubated myotubes with H_2_O_2_ (300 μM for4 h), because H_2_O_2_ is the most stable form of ROS and an established inducer of PARP-1 activity [42]. Myotubes treated with H_2_O_2_ robustly increased PARP-1 activity, as evidenced by higher global protein PARylation (Fig. 4A) without changing PARP-1 mRNA and protein levels (Fig. 4B). H_2_O_2_-treatment also significantly reduced the survival of myotubes in a time-dependent manner (Fig. 4C). However, incubation of myotubes with the PARP-1 inhibitor PJ34 (1 μM for 24 h) before H_2_O_2_ treatment substantially decreased PARP-1 activity (Fig. 4D) and increased the survival of myotubes (Fig. 4E). PARP-1 is the predominant PARP isoform in most tissues and accounts for about 90% of total cellular PARP activity [43]. Therefore, we used RNAi to suppress the level of endogenous PARP-1 in the myotubes (Fig. 4F). As expected, knockdown of PARP-1 decreased both the H_2_O_2_-induced PARP-1 activation (Fig. 4G) and myotube death (Fig. 4H), confirming the role of PARP-1 in ROS-induced myotube death. Next, we studied the impact of over activity of PARP-1 on SIRT-1 function. Myotubes treated with H_2_O_2_ did not exhibit changes in SIRT-1 mRNA and protein levels (Fig. 4I). However, H_2_O_2_-treatment did cause a substantial reduction in SIRT-1 activity, as shown by PGC-1α hyper-acetylation, and pre-incubation of the myotubes with PJ34 or PARP-1-targeting siRNAs blunted this effect (Fig. 4J). Finally, we determined if over-activation of SIRT-1 could rescue myotubes from H_2_O_2_/PARP-1-induced cell death. Treatment of myotubes with the SIRT-1 activator resveratrol (25 μM for 24 h) significantly increased SIRT-1 activity (Fig. 4K) and markedly reduced H_2_O_2_-mediated PARP-1 activity (Fig. 4L) and myotube death (Fig. 4M). These results indicate that SIRT-1 promotes myotube survival and prevents PARP-1-induced myotube death.

### Activated-PARP-1 inhibits SIRT-1 activity by depleting intracellular NAD^+^ levels and poly (ADP-ribosyl)ation

**Figure 5:**
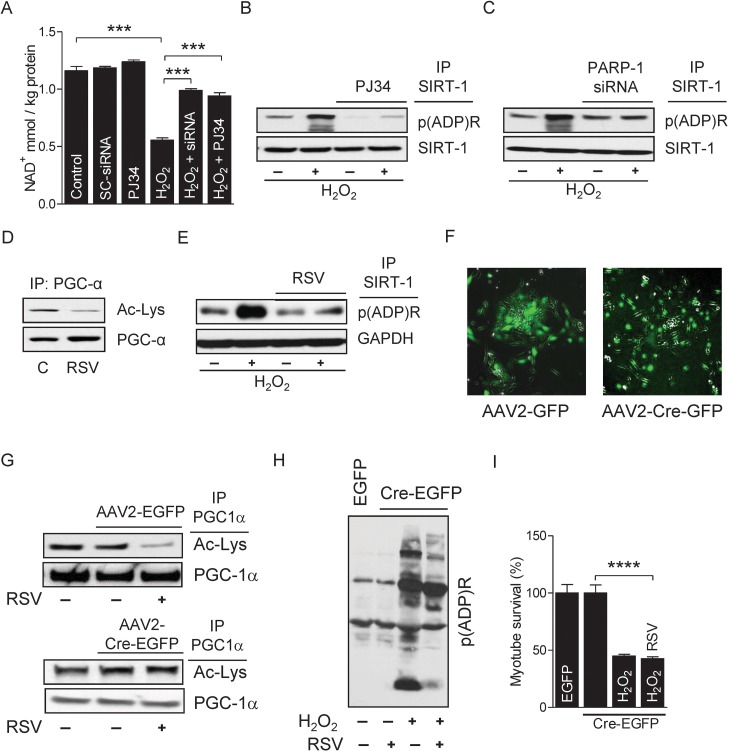
PARP-1 inhibits SIRT-1 activity by PARylation (**A**) NAD^+^ levels were determined in myotubes either in the presence or in the absence of H_2_O_2_ with or without the inhibition of PARP-1 by PJ34 or siRNA. (**B**) NAD^+^ levels were determined in myotubes either in the presence or in the absence of H_2_O_2_ with or without the inhibition of PARP-1 by PJ34 or siRNA. (**B** and **C**) SIRT-1 protein PARylation was determined in total cell lysates from myotubes treated with the conditions similar to ‘A’ by immunoblotting after IP. (**D**) PGC-1α acetylation levels were determined by immunoblotting in total cell lysates from myotubes treated with or without resveratrol. (**E**) SIRT-1 protein PARylation was determined in myotubes either in the presence or in the absence of H_2_O_2_ with or without RSV. (**F**) Infection of myotubes isolated from conditional knockout mice (flox/flox) with AAV2-GFP or AAV2-Cre-GFP to determine SIRT-1 knock-down. (G) PGC-1α acetylation levels were determined in total cell lysate from myotubes infected with either AAV2-GFP or AAV2-Cre-GFP in the presence or absence of RSV by immunoblotting after IP. Global cellular protein PARylation (**H**) and percentage of myotube survival (**I**) were determined in either AAV2-GFP or AAV2-Cre-GFP infected myotubes in the presence or in the absence of H_2_O_2_ or RSV. AAV, adeno-associated virus; GFP, green fluorescence protein; IP, immunoprecipitation; RSV, resveratrol.

We next studied the molecular mechanism by which over activation of PARP-1 inhibits SIRT-1 function. Since SIRT-1 and PARP-1 share the same nuclear NAD^+^ pool, it is possible that one enzyme may influence the other's activity through competition for NAD^+^ [35]. Therefore, we measured the intracellular NAD^+^ content in myotubes treated with H_2_O_2_ and found that the treatment significantly decreased intracellular NAD^+^ content; however, inhibition of PARP-1 by either PJ34 or siRNAs blocked H_2_O_2_–induced NAD^+^ depletion (Fig. 5A). Although the depletion of intracellular NAD^+^ levels by PARP-1 inhibits SIRT-1 activity, it is not known whether PARP-1 also inhibits SIRT-1 activity in skeletal muscles by PARylation. Thus, we conducted IP assays using nuclear extracts from H_2_O_2_-treated myotubes with an antibody specific for PARylated proteins followed by immunoblot assays with an anti-SIRT-1 antibody. As shown in F igure 5B, H_2_O_2_ treatment had a marked increase in the level of SIRT-1 PARylation (Fig. 5B). Likewise, IP assays with an antibody specific for SIRT-1 followed by immunoblot assays with an anti-PAR antibody confirmed the H_2_O_2_-induced PARylation of SIRT-1 (Fig. 5C). However, reatment of myotubes with PJ34 or PARP-1-targeted siRNAs reversed the effect of PARP-1 on SIRT-1 PARylation (Fig. 5B and C), indicating that over-activation of PARP-1 inhibits the activity of SIRT-1 by both depleting intracellular NAD^+^ and PARylating SIRT-1. Next, we determined whether over activation of SIRT-1 could rescue myotubes from H_2_O_2_/PARP-1-induced cell death. Towards this end, we treated myotubes with resveratrol, which significantly increased SIRT-1 activity (Fig. 5D). Activation of SIRT-1 by resveratrol reduced PARP-1-mediated SIRT-1 PARylation (Fig. 5E). To explore the role of SIRT-1 in myotube survival, we isolated primary myoblasts (i.e. satellite cells) from SIRT-1 conditional (floxed) knockout mice (SIRT-1^loxp/loxp^) and infected these cells with AAV transducing either GFP or Cre (Fig. 5F). Infection of the floxed primary myoblasts with Cre, but not with GPF significantly reduced the activity of SIRT-1 (Fig. 5G). Activation of PARP-1 in AAV-Cre-infected myotubes by H_2_O_2_ significantly increased global protein PARylation (Fig. 5H) and decreased the survival of myotubes (Fig. 5I); however, treatment of these myotubes with resveratrol did not reverse the effect of H_2_O_2_ (Fig. 5H and I). These results indicate that H_2_O_2_-induced PARP-1 activation induces myotube death via inhibition of SIRT1 activity due to depletion of intracellular NAD^+^ and increased SIRT-1 PARylation.

## DISCUSSION

Skeletal muscle undergoes a profound age-related deterioration called sarcopenia, which is characterized by a marked decline in muscle mass and function. Although the pathogenesis of sarcopenia is complex, ROS accumulation stemming from mitochondrial dysfunction plays a key role in this process. PARP-1 is a central mediator of the response to cellular stress caused by physiological stressors, such as ROS and inflammation. While the basal activity of PARP-1 is necessary to maintain genome integrity and cellular homeostasis in response to oxidative stress, over-activation of PARP-1 induces a skeletal muscle decline that is more common in aged individuals than in younger people. Therefore, it is possible that regulation of skeletal muscle protein PARylation by PARP-1 would be different in aged people than that in younger individuals. Here, we demonstrate that in aged mice, exercise over-activates PARP-1 via GCN5-dependent acetylation in skeletal muscle and that activated-PARP-1 depletes cellular NAD^+^ levels, thereby inhibiting SIRT-1 activity that resulted in reduced mitochondrial biogenesis and metabolism, and increased the onset of fatigue and reduced the ability to produce and work. In contrast, SIRT1 has the ability to bind with and deacetylate PARP-1, and as a result of reduced PARP-1 activity, there is an increase in mitochondrial content and biogenesis in skeletal muscle from young mice. Interestingly, inhibition of PARP-1 in aged mice reduced the exercise-induced over activation of PARP-1 and promoted mitochondrial biogenesis via the SIRT-1/PGC-1α pathway, which resulted in an increased onset of fatigue an a decreased ability to produce total muscle work, suggesting that activation of SIRT-1 and/or inhibition of PARP-1 would be an effective way to improve muscle function in aging. As PJ34 delayed the onset (although not the extent) of fatigue, and it also improved the capacity to sustain the higher forces over the duration of the 360 contractions in our experiments, the ability to produce and sustain ATP production and delivery from the mitochondria to the muscles was presumably better when PARP-1 was inhibited. This speculation is consistent with observations of greater signaling for mitochondrial biogenesis (which would be the primary source of ATP generation) when PARP-1 was inhibited. It would have been interesting to test if the PJ34 would have markedly lowered the muscle fatigue rate as compared to control muscles throughout a sustained effort, if the muscles in both treatment groups had been required to maintain an identical absolute submaximal load over the course of the experiment (although this was not tested in the current study).

SIRT-1 has emerged as a major therapeutic target for aging and age-associated diseases, including sarcopenia [44, 45]. Therefore, understanding how oxidative stress modulates SIRT-1 is crucial to unraveling the mechanisms underlying sarcopenia. SIRT-1 has also been shown to protect skeletal muscle against ROS-induced muscle damage [46]. One interesting approach to ameliorating age-related skeletal muscle disorders would be to elevate intracellular NAD^+^ content, thereby activating the NAD^+^-dependent enzyme SIRT-1. In line with this, several studies have shown that increasing NAD^+^ levels in old mice restored mitochondrial function and decreased lactate production, reversing a pseudo-hypoxic state [47], which may be mTOR-dependent [48, 49]. In conjunction with increasing cellular NAD^+^ content, hindering other NAD^+^-dependent proteins would also be expected to enhance SIRT-1 activity. For example, in the present study, we showed *in vitro* and *in vivo* that inhibition of PARP-1, a major cellular NAD^+^ consumer, increased cellular NAD^+^ levels, which increased SIRT-1 activity, thereby augmenting mitochondrial content and biogenesis. We also showed that over-activation of PARP-1 by H_2_O_2_ increased global protein PARylation and depleted intracellular NAD^+^ content leading to myotube death. This suggests that one enzyme may influence the other's activity through competition for NAD^+^ and that a tight regulation of PARP-1 activity is important for cell survival. Our present work supports this concept by showing how the attenuation of PARP-1 increased intracellular NAD^+^ levels and enhanced SIRT-1 activity. This effect prompted the deacetylation and activation of the key metabolic transcriptional regulator PGC-1α, leading to increased mitochondrial content and biogenesis. Our data provide strong support for the idea that in aging skeletal muscles, over-activation of PARP-1 limits NAD^+^ availability for SIRT-1 function. This concept derives from the variation in the K_M_ and k_cat_/K_M_ of both enzymes for NAD^+^, which indicates that PARP-1 is more rapid and a more efficient NAD^+^ consumer than is SIRT1 [50]. Hence, it is possible that PARP-1 activity modulates NAD^+^ and this regulates SIRT1 function. While earlier data have reported that exercise increases SIRT1-activity [51]and other studies had speculated on a link between PARP-1 and SIRT-1 activities [32, 35], our study expands the consequences of this link to sarcopenia. In the present study, we demonstrated that the exercise-induced activation of PARP-1 significantly reduced SIRT-1 activity due to depletion of cellular NAD^+^ content and as a result increased skeletal muscle fatigue in aged mice. Interestingly, *in vivo* inhibition of PARP-1 by PJ34 blocked PARP-1 activity resulting in higher SIRT-1/PGC-1α activity and mitochondrial content and biogenesis in skeletal muscle from aged mice. More importantly, inhibition of PARP-1 significantly improved muscle performance in aged mice. Moreover, activation of SIRT-1 by resveratrol treatment mimicked the effects of *in vivo* PARP-1 inhibition in myotubes. In agreement with the findings of our present study, our earlier results have shown that resveratrol appears to have modest therapeutic benefits for improving muscle mass in aged animals [38, 52, 53]. These results suggest that the activation of SIRT-1 by either modulating NAD^+^ availability or inhibiting other NAD^+^ consumers, such as PARP-1, could be an alternative means to activate SIRT-1 and perhaps to ameliorate, at least in part, the pathogenicity of sarcopenia.

Skeletal muscle abundantly expresses PARP-1, especially in response to oxidative stress [32]. In the present study, we demonstrated the mechanism of PARP-1 regulation in skeletal muscle. More precisely, treatment of myotubes with H_2_O_2_ or exercise in skeletal muscle, especially in aged mice, robustly activated PARP-1, as evidenced by enhanced PARylation of skeletal muscle proteins in addition to an enhanced acetylation of PARP-1 protein, indicating that acetylation of PARP-1 had indeed contributed to the increased PARP-1 activity. This is consistent with previous studies, which reported that treatment of cardiomyocytes with H_2_O_2_ increased both the acetylation level and the activity of PARP-1 [36]. Furthermore, data from our present study suggest that skeletal muscles from young and aged mice differentially regulate protein PARylation and that PARP-1 might be one of the downstream targets of the stress stimuli that initiate sarcopenia. Although many members of class I HDACs deacetylate PARP-1, in the present study, we found that SIRT-1 was also capable of deacetylating PARP-1, which is consistent with the results of a previous study in cardiomyocytes [37]. Our *in vitro* study further confirm that a deletion of the full catalytic core domain of SIRT-1, which eliminated its deacetylase activity, did not affect its binding to PARP-1; however, the deletion failed to suppress skeletal muscle protein PARylation. Introduction of resveratrol had no effect on the level of protein PARylation, indicating that the catalytic activity, and not the protein binding ability of SIRT-1, was necessary for blocking PARP-1 activity. These results also corroborate that deacetylation of PARP-1 in skeletal muscles is unique to SIRT-1, because the deletion of exon 4 of the SIRT-1 gene is unique to the SIRT-1 catalytic core domain rather than to other members of sirtuin family. This observation provides evidence for SIRT-1-dependent inactivation of PARP-1 in muscle. In line with our current findings, previous studies have shown that the acetyltransferase p300/CBP acetylates PARP-1 and increases its ability to regulate NF-κB-dependent gene transcription [39]. Another study has shown that the acetyltransferase PCAF acetylates PARP-1 [37]. Although, these two studies identified different lysine residues with different rates of acetylation by p300/CBP and PCAF, one of the PARP-1 fragments (aa 477 to 525 segment) was highly acetylated by both p300/CBP and PCAF. This suggests that PCAF and p300/CBP may have different preferences for lysine residues within PARP-1. In the present study, we identified GCN5 as a new acetyltransferase and activator of PARP-1. With a series of IP assays, we showed that in young mice, electrically evoked isometric exercise significantly decreased the acetylation level of PARP-1 due to an increased association with SIRT-1 and dissociation from GCN5. In contrast, the exercise-induced acetylation of PARP-1 was greatly increased in aged mice due to decreased association with SIRT-1 and increased association with GCN5. These results indicate that dysregulation of SIRT-1 may be a primary mechanism that regulates the over-activation of PARP-1 in the skeletal muscles of aged mice. Furthermore, it was logical to anticipate that GCN5 could act as an acetyltransferase of PARP-1, because GCN5 has been shown to acetylate other skeletal muscle proteins in response to a variety of stimuli [54] and it is 70% identical to PCAF [55], which is a p300/CREB binding protein/associated factor. Therefore, it is reasonable to expect that GCN5, PCAF, and p300/CBP may control PARP-1 activity differently in different cellular and physiological conditions. It will be interesting to determine which lysine residues within PARP-1 are acetylated by GCN5 in skeletal muscle.

**Figure 6:**
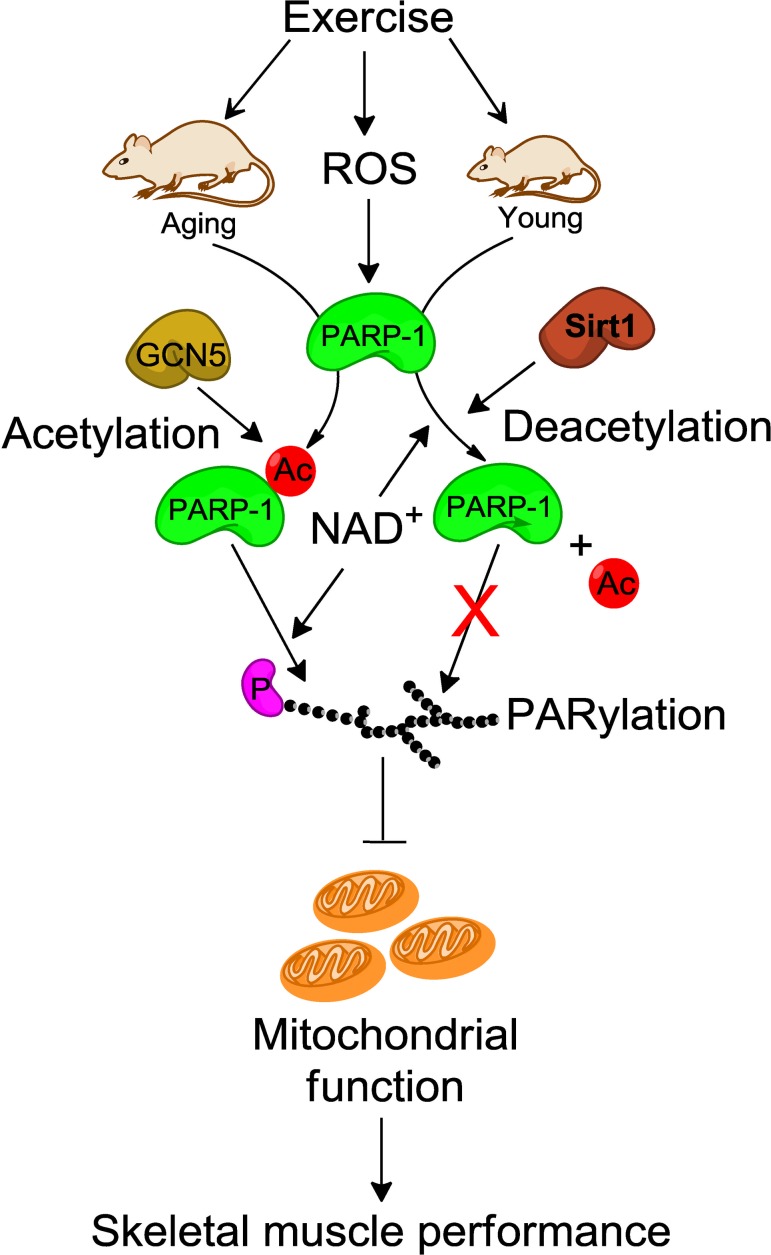
Schematic illustration of how SIRT-1 may protect skeletal muscle from PARP-1-induced muscle fatigue In young mice, SIRT-1 inhibits the exercise-induced PARP-1 activity by deacetylation-dependent mechanism and as a result increases skeletal muscle performance via enhanced mitochondrial biogenesis. In contrast, dysregulation of SIRT-1 in skeletal muscle from aged mice reduces skeletal muscle performance due to higher PARP-1 activity via GCN5 mediated acetylation. ROS, reactive oxygen species; NAD, nicotinamide adenine dinucleotide; Ac, acetylation; P, protein.

Many lines of evidence have shown opposing roles of PARP-1 and SIRT-1 for the same target. For example, while PARP-1 increases p53 nuclear translocation and its transcriptional activity via PARylation, SIRT-1 deacetylates p53 and inhibits its transcriptional activity [56, 57]. Similarly, PARP-1 and SIRT-1 regulate other targets such as NF-κB, FOXO, and Ku70 [57-59] in opposite directions. Thus, in order for the activity of one of the two enzymes to pre-dominate, it is necessary to inhibit the activity of the other. The data in the present study demonstrate that both PARP-1 and SIRT-1 have the capability to counterbalance each other's activity in young and aged mice. The model proposed in Fig. 6 depicts how SIRT-1 and PARP-1 may regulate each other's activity in skeletal muscle between young and aged mice to affect mitochondria abundance thereby modulating muscle fatigue and potentially impacting sarcopenia.

In summary, we demonstrated that ROS-induced PARP-1 activation decreased myotube survival via depleting cellular NAD^+^ levels, and that depletion inhibits SIRT-1 activity. We also demonstrated a new mode of PARP-1 activation by GCN5-mediated acetylation in skeletal muscle. SIRT-1 has the ability to physically bind to PARP-1 and deacetylate it, and that effect results in suppression of PARP-1 enzymatic activity in skeletal muscle from young mice following exercise. This protective role of SIRT-1 was suppressed in skeletal muscle of aged mice, but this suppression was reversed by PARP-1 inhibition. These data provide strong evidence for the existence of a functional interplay between PARP-1 and SIRT-1 and give novel insights into the modulation of skeletal muscle fatigue by SIRT-1 in sarcopenia.

## METHODS

### Animals

The Institutional Animal Care and Use Committee from the West Virginia University School of Medicine approved all experimental procedures. A total of 34 mice were used in these experiments. These include 3 month old young (n=16), and 26 months old aged (n=18) male C57BL/6J mice (The Jackson Laboratory, Bar Harbor, ME). All mice were kept in a temperature-controlled room on a 12-h light/dark cycle, with 60% humidity, and food and water *ad libitum*. Mice were anesthetized with 2% isoflurane gas using a small animal anesthetic system (Surgivet Anesco Inc., Waukesha, WI.) before electrically-evoked contractions (exercise) or terminal surgery.

### Electrically-evoked isometric contractions

*In situ* electrically stimulated isometric contractions were conducted on a custom-built mouse dynamometer as described previously [9]. Briefly, mice were anesthetized with a mixture of oxygen (97%) and isoflurane gas (3%) and placed on a plate that was heated to 37°C. The right ankle was positioned at 90°C of flexion and was aligned with the axis of rotation of the servomotor (Model 6350* 350; Cambridge Technology, Scientific, Aurora, ON, Canada). The foot was secured to the footplate that was connected to the servomotor. Commercially available software (Dynamic Muscle Control; Aurora Scientific, Aurora, ON, Canada) was used to control the servomotor providing for the angular position of the foot.

Muscle contractions of the plantar flexor muscles were stimulated via subcutaneous platinum electrodes that were placed on either side of the tibial nerve near the popliteal fossa. Electrode placement was tested via a short stimulation of the nerve to cause plantar flexion twitches. When stimulated, the foot plantar flexed without any visible appearance of eversion, or inversion, of the foot. Twenty electrically evoked (10-V, 100-Hz, 200 μs pulses) isometric contractions of the plantar flexor muscle group were obtained in one limb. Each contraction train lasted for 3 s, and a 10-s recovery period occurred between subsequent contractions. The contralateral limb served as the intra animal control. Muscle functional data were collected as a force×time curve during isometric contractions for each session and values were normalized to each animal's body weight. Muscle fatigue data were assessed over 360 contractions at 40Hz (0.3s duration with 200 μs pulses). The fatigue index (expressed as percent of the starting force) was calculated as: [the first contraction – desired contraction)/first contraction x 100]. The contractile data were analyzed offline (Dynamic Muscle Analysis software; Aurora Scientific).

### Cell Culture

Isolation of primary myoblasts from SIRT-1^flox/flox^ mice was performed as described previously [60]. Before starting the experiments, myoblasts from passages 5 and 6 were cultured in growth medium (GM; DMEM containing 20% fetal calf serum, 100 units/ml penicillin, and 100 μg/ml streptomycin), and differentiation was induced by replacing GM with differentiation medium (DM; GM containing 2% horse serum instead 20% fetal calf serum) when they were 70-80% confluent. Myotubes maintained in DM for four days were used in all experiments.

### siRNA transfection

For siRNA-mediated knockdown studies, myotubes were transfected with 500 pmol of siRNA specific for mouse PARP-1 or nonspecific siRNA (Santa Cruz Biotechnology). RNA transfection studies were performed with Lipofectamine RNAi MAX (Invitrogen) according to the manufacturer's instructions. After 8 h, the transfection medium was replaced with DM. Subsequent assays were conducted 24 to 48 h after transfection.

### Cell survival assay

Fresh DM was added to both treated and non-treated myotubes cultured in 24-well plates followed by incubation with 100 μl MTT solution (10 mg/ml in PBS) for 4 h in a cell culture incubator. One hundred microliters of lysis buffer (20% SDS in 50% dimethyl formamide, pH 4-7) was added to each well, and then the plates were further incubated for 16 h in a cell culture incubator. The absorbance was read at 570 nm using a microplate reader (DynexTechnologies Limited, Worthing, West Sussex, UK). Wells containing DM without myotubes were included as a blank.

### Cellular NAD^+^

NAD^+^ was estimated colorimetrically using an NAD/NADH assay kit (Abcam, Cambridge, MA) as described previously [61]. The standard was prepared according to the manufacturer's protocol. The NAD/NADH ratio was calculated using the formula: NADt (NAD and NADH) — NADH/NADH and was expressed as mM/kg protein.

### Reverse transcription and quantitative PCR

Real-time RT-PCR was performed as described previously (Mohamed *et al*. 2013). The relative amounts of amplified transcripts (2^−△CT^) were estimated by the comparative C_T_ (−△CT) method and normalized to an endogenous reference (GAPDH) relative to a calibrator. All PCR products were verified on an agarose gel stained with ethidium bromide to discriminate between the correct amplification products and potential primer dimers. The primers used in this study are described previously [61].

### Immunoblots

Total cell lysate extraction from either myotubes or skeletal muscles and westernblot were performed as described previously [60]. Anti-p(ADP)R (sc-56198) and anti-PGC-1α(sc-13067) were purchased from Santa Cruz Biotechnology. Anti-PARP-1 (9542), anti-GAPDH (5174), anti-acetylated lysine (9441) and anti-GCN5 (3305) were purchased from Cell Signaling.

### *In vitro* pull-down assay

Protein-protein interactions and protein acetylation levels were determined by immunoprecipitation (IP) using 150 μg protein and A/G-agarose beads as described previously [60, 61].

Statistical analysis. The results are expressed as the means ± SEM. Comparisons among different groups were performed by one-way ANOVA followed by Bonferroni post-testing. Paired data were evaluated by Student t test. A *P* value of < 0.05 was considered statistically significant. Each experiment was repeated at least three times using three different mice.
